# Identification of MICALL2 as a Novel Prognostic Biomarker Correlating with Inflammation and T Cell Exhaustion of Kidney Renal Clear Cell Carcinoma

**DOI:** 10.7150/jca.66922

**Published:** 2022-01-16

**Authors:** Wenfeng Lin, Wenwei Chen, Jisheng Zhong, Hideo Ueki, Abai Xu, Masami Watanabe, Motoo Araki, Chunxiao Liu, Yasutomo Nasu, Peng Huang

**Affiliations:** 1 Department of Urology, Okayama University Graduate School of Medicine, Dentistry and Pharmaceutical Sciences, Okayama, Japan.; 2 Department of Urology, Zhujiang Hospital, Southern Medical University, Guangzhou, China.; 3 Department of Urology & Department of Kidney Transplantation, The First Affiliated Hospital, Wenzhou Medical University, Wenzhou, China.; 4 School of Medicine, Xiamen University, Xiamen, China.; 5 Center for Innovative Clinical Medicine, Okayama University Hospital, Okayama, Japan.; 6 Okayama Medical Innovation Center, Okayama University, Okayama, Japan.

**Keywords:** MICALL2, biomarker, inflammation, T cell exhaustion, kidney renal clear cell carcinoma

## Abstract

**Purpose:** The interplay of inflammation and immunity affects all stages from tumorigenesis to progression, and even tumor response to therapy. A growing interest has been attracted from the biological function of MICALL2 to its effects on tumor progression. This study was designed to verify whether MICALL2 could be a prognostic biomarker to predict kidney renal clear cell carcinoma (KIRC) progression, inflammation, and immune infiltration within tumor microenvironment (TME).

**Methods:** We firstly analyzed MICALL2 expressions across 33 cancer types from the UCSC Xena database and verified its expression in KIRC through GEPIA platform and GEO datasets. The clinicopathological characteristics were further analyzed based on the median expression. Kaplan-Meier method, univariate and multivariate analyses were applied to compare survival outcomes. ESTIMATE and CIBERSORT algorithms were performed to assess immune infiltration, and a co-expression analysis was conducted to evaluate the correlation between MICALL2 and immunoregulatory genes. Enrichment analysis was finally performed to explore the biological significance of MICALL2.

**Results:** MICALL2 was highly expressed in 16 types of cancers compared with normal tissues. MICALL2 expression increased with advanced clinicopathological parameters and was an independent predictor for poor prognosis in KIRC. Moreover, MICALL2 closely correlated with inflammation-promoting signatures and immune infiltration including T cell exhaustion markers. Consistently, MICALL2 involved in the regulation of signaling pathways associated with tumor immunity, tumor progression, and impaired metabolic activities.

**Conclusion:** MICALL2 can function as a prognostic biomarker mediating inflammation, immune infiltration, and T cell exhaustion within the microenvironment of KIRC.

## Introduction

Tumor microenvironment (TME), a fertile soil for cancer progression, is composed of immune cells, bone marrow-derived inflammatory cells, fibroblasts, various signal molecules, extracellular matrix, and blood vessels 1. As the basic characteristics of TME, immunity and inflammation involve in all stages of tumor development, but their relationship is still vague so far 2, 3. The anti-tumor and tumor-promoting immune and inflammatory mechanisms coexist in developing tumor, which will affect the choice of treatment strategies and the efficacy at different stages 4. Moreover, there is only a small portion of patients who respond well to immunotherapy 5. Therefore, it remains a challenge to explore appropriate biomarkers for predicting which cohort will benefit most from immunotherapy. In recent years, researchers have developed the ESTIMATE algorithm and CIBERSORT tool to assess immune infiltration in TME 6, 7. The single sample Gene Set Enrichment Analysis (ssGSEA) has also been applied to quantify various immune signatures, such as check-point, inflammation-promoting 8, 9. All these methods will help to find the potential biomarkers to predict immune and inflammatory response with which we can better select those responding well to immunotherapy or the combined therapy.

Accumulating evidence has shown that the molecule interacting with CasL (MICAL) family participates in cytoskeleton dynamics, which is composed of two MICAL-L homologues (MICAL-L1, -L2) and three MICALs (MICAL1-3) 10. MICAL-L2, with an alternative name as molecule interacting with CasL-like 2, is encoded by MICALL2 gene 11. Previous studies have demonstrated the crucial role of MICALL2 in cytoskeleton reorganization, tight junction assembly, and neurite outgrowth 12-14. Interestingly, MICALL2 also regulates the epithelial cell adhesion, repulsion, and even scattering 15, 16. Therefore, in recent years, more attention has been gradually attracted from its biological function to the effects of MICALL2 on cancer progression. Silencing of MICALL2 can suppress the invasion, metastasis, and proliferation of ovarian cancer via regulating canonical Wnt/β-catenin pathway and epithelial-mesenchymal transition (EMT) 17. Gastric cancer cell migration is also potentiated through MICALL2 enhancing the stability of epidermal growth factor receptor (EGFR) 18. In addition, MICALL2 binds to c-Myc and reduces its ubiquitin-dependent degradation, thus promoting the cell proliferation of non-small cell lung cancer (NSCLC) 19. However, it remains poorly understood whether MICALL2 can be developed as an ideal biomarker to predict cancer progression and patient prognosis. Furthermore, it remains to be verified whether MICALL2 is correlated with immune infiltration and inflammation in TME.

**Figure 1:**
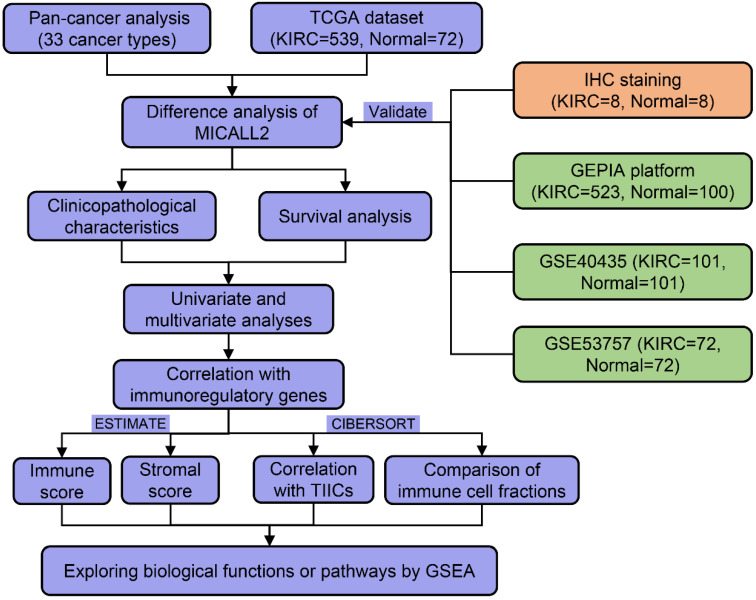
The flow process diagram of MICALL2 bioinformatics analysis.

As shown in the flow process diagram **(Figure 1)**, we evaluated MICALL2 differential expressions across 33 cancer types and their corresponding normal tissues. Then we conducted the preliminary exploration on the role of MICALL2 expression in pan-cancers. Based on the significant results, we hypothesized that MICALL2 expression had close relation to patient prognosis, immune infiltration, and tumor progression in kidney renal clear cell carcinoma (KIRC) which was mainly investigated in the follow-up bioinformatics analyses. In addition, the potential biological functions or pathways of MICALL2 were screened using the gene set enrichment analysis (GSEA). Finally, two Gene Expression Omnibus (GEO) datasets were introduced for further validation analysis. All these findings revealed that MICALL2 could serve as a prognostic biomarker to predict the inflammatory and immune response within the TME, providing theoretical support for the precision treatment of KIRC.

## Material and methods

### Data Acquisition

For pan-cancer analysis, the clinicopathological and transcriptome data containing 33 cancer types were provided by UCSC Xena database (http://xena.ucsc.edu). KIRC dataset with normal kidney tissues (N) = 72, and KIRC samples (T) = 539, was provided by TCGA database (https://portal.gdc.cancer.gov). The inclusion criteria can be found on the website (https://www.ncbi.nlm.nih.gov/projects/gap/cgi-bin/study.cgi?study_id=phs000178.v3.p3). GSE53757 (N = 72, T = 72) and GSE40435 (N = 101, T = 101) datasets were obtained from Gene Expression Omnibus (GEO) database (https://www.ncbi.nlm.nih.gov/geo/).

### Gene Expression Profiling Interactive Analysis (GEPIA)

GEPIA platform (http://gepia.cancer-pku.cn/), was used to compare MICALL2 expression levels between normal samples and tumor samples by matching TCGA normal and Genotype-Tissue Expression (GTEx) data (|log_2_FC| Cutoff =1, P-value Cutoff = 0.01).

### Immunohistochemistry (IHC) staining of clinical KIRC samples

We analyzed 8 pairs of KIRC and adjacent samples from the First Affiliated Hospital of Wenzhou Medical University. After 65℃ baked for 2 h, the slices then underwent dewaxing and antigen retrieval. 3% hydrogen peroxide was used to inactivate endogenous enzymes for 10 min. Following PBS washing and block by bovine serum albumin, the slices underwent 4℃ overnight incubation with MICALL2 antibody (#bs-18936R, Bioss Inc) 37℃ 30 min incubation with the secondary antibody. Next, DAB color rendering and hematoxylin redye were performed for 5-10 min and 3 min, respectively. The measurement of integrated optical density (IOD) was conducted by Image Pro Plus 6.0 image software.

### Survival Analysis

Based on MICALL2 median expression, the patients were divided into high-MICALL2 and low-MICALL2 groups. Kaplan-Meier (K-M) method was introduced to analyze Overall Survival (OS), Disease-Specific Survival (DSS), Progression-Free Interval (PFI) by R packages “survival” and “survminer”. Cox regression was further applied for the impact assessment of clinicopathological factors (pathological grade, clinical stage, T classification, and M classification, age, gender), and MICALL2 expression on survival based on univariate and multivariate analyses.

### MICALL2 Expression and Immunity

The ESTIMATE algorithm 7 was applied to assess immune infiltration (ImmuneScore, StromalScore, ESTIMATEScore, and TumorPurity), followed by the comparisons of immune infiltration between low-MICALL2 and high-MICALL2 expression groups by R software packages “estimate” and “limma”. The tool CIBERSORT 6 was applied for integrative analysis between MICALL2 expression and 22 tumor-infiltrating immune cells (TIICs). The ssGSEA using R package “GSVA” was applied for the quantification of 11 immune signatures, including MHC class I, APC co inhibition, chemokine receptors (CCR), APC co stimulation, T cell co-inhibition, check-point, T cell co-stimulation, inflammation-promoting, parainflammation, Type I IFN response, and Type II IFN response 20. The R packages “reshape2” and “RColorBrewer” were applied to perform a co-expression analysis of MICALL2 and immunoregulatory genes associated with major histocompatibility complex (MHC), immunosuppression, immune activation, chemokines, and chemokine receptors 21.

### The Biological Significance of MICALL2 Expression in KIRC

The GSEA software (version 4.1.0) was applied to perform functional enrichment analysis of MICALL2 in KIRC. As the reference, the annotated “c2.cp.kegg.v7.4.symbols.gmt” was introduced to explore the potential biological pathways that MICALL2 may regulate in KIRC.

### GEO Datasets Validation Analysis

The GSE53757 and GSE40435 datasets were used to validate MICALL2 expression difference between KIRC samples and normal tissues. For GSE53757 dataset, the differentially expressed genes (DEGs) were identified to screen the biological functions and signaling pathways between low-MICALL2 and high-MICALL2 expression samples by “limma” package (P-value <0.05; |logFC| >0.5). The enrichment tools such as Gene Ontology (GO) term, and Kyoto Encyclopedia of Genes and Genomes (KEGG) were then introduced to analyze the DEGs using R packages clusterProfiler, enrichplot, and ggplot2.

### Statistical Analysis

The comparisons of between-group MICALL2 expressions were conducted by Wilcoxon test. The correlation concerning MICALL2 expression was analyzed by Spearman's correlation test. K-M method, univariate and multivariate analyses were introduced to compare survival outcomes. Statistical analyses were performed with GraphPad Prism (version 8.3) and R software (version 4.1.0). Statistical differences were confirmed when P values were less than 0.05.

## Results

### Differential MICALL2 expression between normal and tumor tissue samples

**Figure 2:**
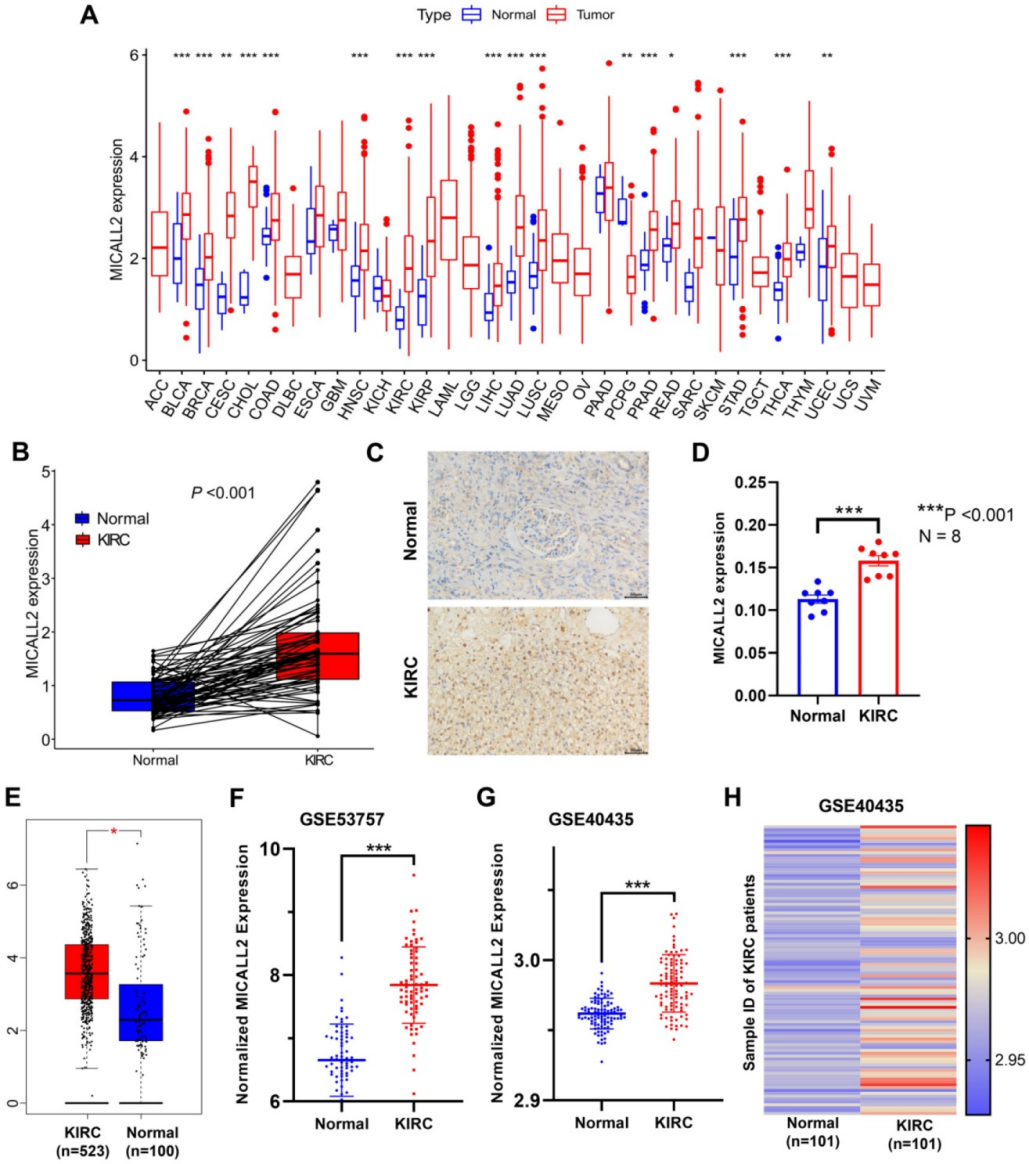
** Differential expression of MICALL2 between normal and tumor tissue samples.** MICALL2 expression in pan-cancers and their corresponding normal samples from UCSC Xena database. **(B)** Paired comparison analysis of MICALL2 expression between KIRC tissues and their matched normal tissues from TCGA database. **(C-D)** The immunohistochemical staining of KIRC samples and the quantitative analysis of MICALL2 shown as average optical density (IOD of positive area/tissue area under visual field). Scale bar=50μm. **(E-G)** The validation of differential MICALL2 expression by GEPIA platform, GSE53757, and GSE40435 datasets. **(H)** The heatmap of MICALL2 expression by pairwise comparison in GSE40435 dataset. (*P <0.05, **P <0.01, ***P <0.001).

UCSC Xena database was introduced to analyze the differential expression of MICALL2 across 33 cancer types and their corresponding normal tissues (**Figure 2A**). Our results suggested that MICALL2 was highly expressed in 16 types of cancers (BLCA, BRCA, CESC, CHOL, COAD, HNSC, KIRC, KIRP, LIHC, LUAD, LUSC, PRAD, READ, STAD, THCA, and UCEC) compared with their corresponding normal tissues. In contrast, MICALL2 levels were downregulated in PCPG tissues relative to normal tissues. We next preliminarily explored the role of MICALL2 expression in pan-cancers. Based on the significant results, we hypothesized that MICALL2 expression had close relation to patient prognosis, tumor progression, and immune infiltration in KIRC which was mainly investigated in the follow-up bioinformatics analyses. Then we performed paired comparison analysis on KIRC dataset from TCGA database, showing that MICALL2 was upregulated in KIRC samples compared with paired normal samples (**Figure 2B**). Consistently, higher MICALL2 expressions in KIRC than normal tissues were validated by IHC staining (**Figure 2C-2D**), GEPIA platform (**Figure 2E**), GSE53757 (**Figure 2F**), and GSE40435 datasets (**Figure 2G**). The heatmap by pairwise comparison also presented the significantly differential expression of MICALL2 in GSE40435 dataset (**Figure 2H**).

### High MICALL2 levels associate with advanced clinicopathological characteristics in KIRC patients

**Figure 3:**
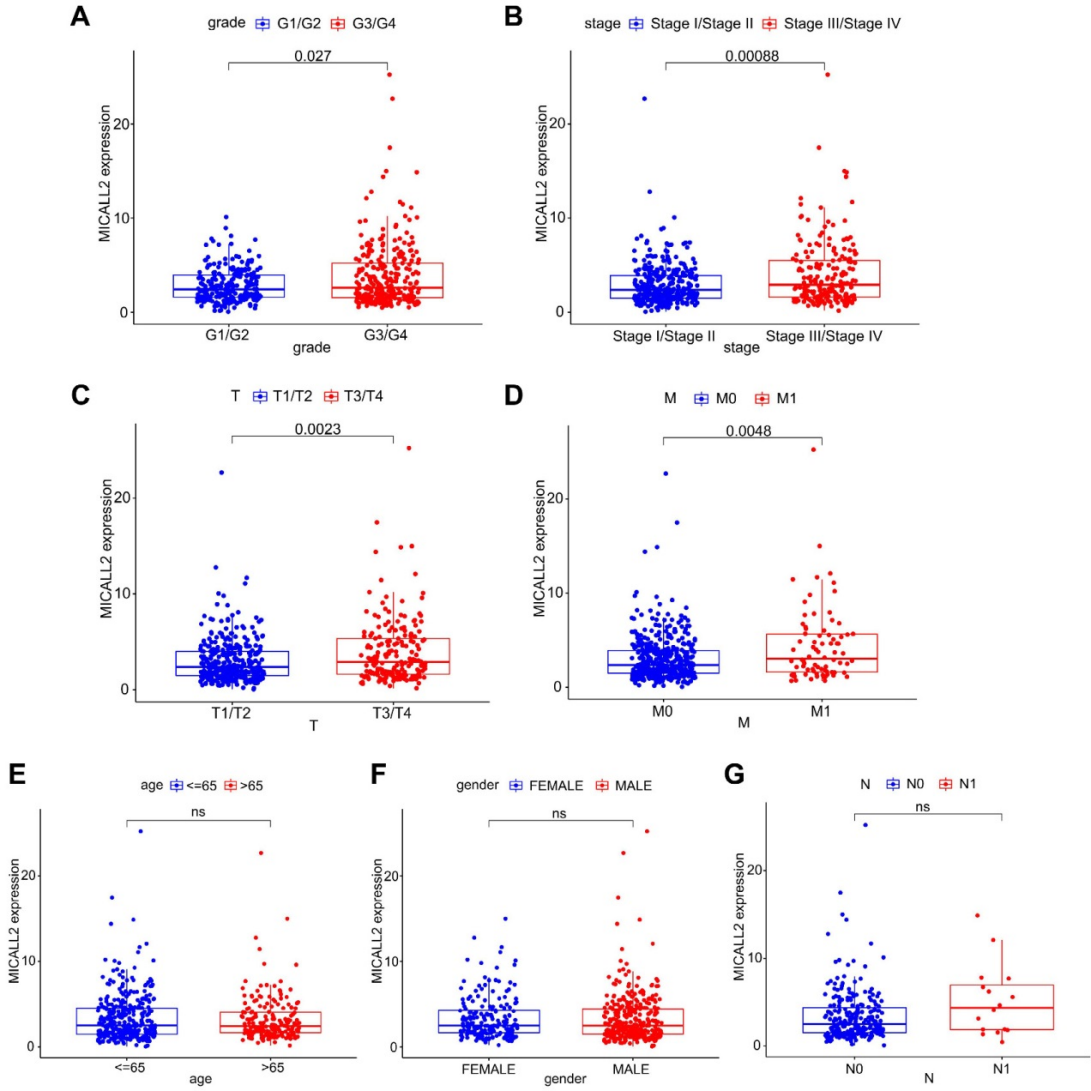
** The correlation of MICALL2 expression with clinicopathologic characteristics in KIRC.** MICALL2 expression was positively correlated with **(A)** pathological grade, **(B)** clinical stage, **(C)** T classification, **(D)** M classification. But no statistical difference in MICALL2 expression was found **(E)** between ≤65 and >65, **(F)** between Female and Male, **(G)** between N0 and N1.

Differential MICALL2 expression was further examined according to the clinicopathological characteristics including age, gender, pathological grade, clinical stage, T classification, M classification, and N classification (**Table 1**). MICALL2 expressions in groups of G3/G4, Stage III/Stage IV, T3/T4, and M1 were significantly higher than those in groups of G1/G2, Stage I/Stage II, T1/T2, and M0, respectively (**Figure 3A-3D**). But there was no statistical difference in MICALL2 expression between ≤65 and >65, between Female and Male, between N0 and N1 (**Figure 3E-3G**). Therefore, our findings revealed that MICALL2 expression was positively correlated with tumor progression.

### MICALL2 is an independent predictor for poor prognosis in KIRC patients

**Figure 4:**
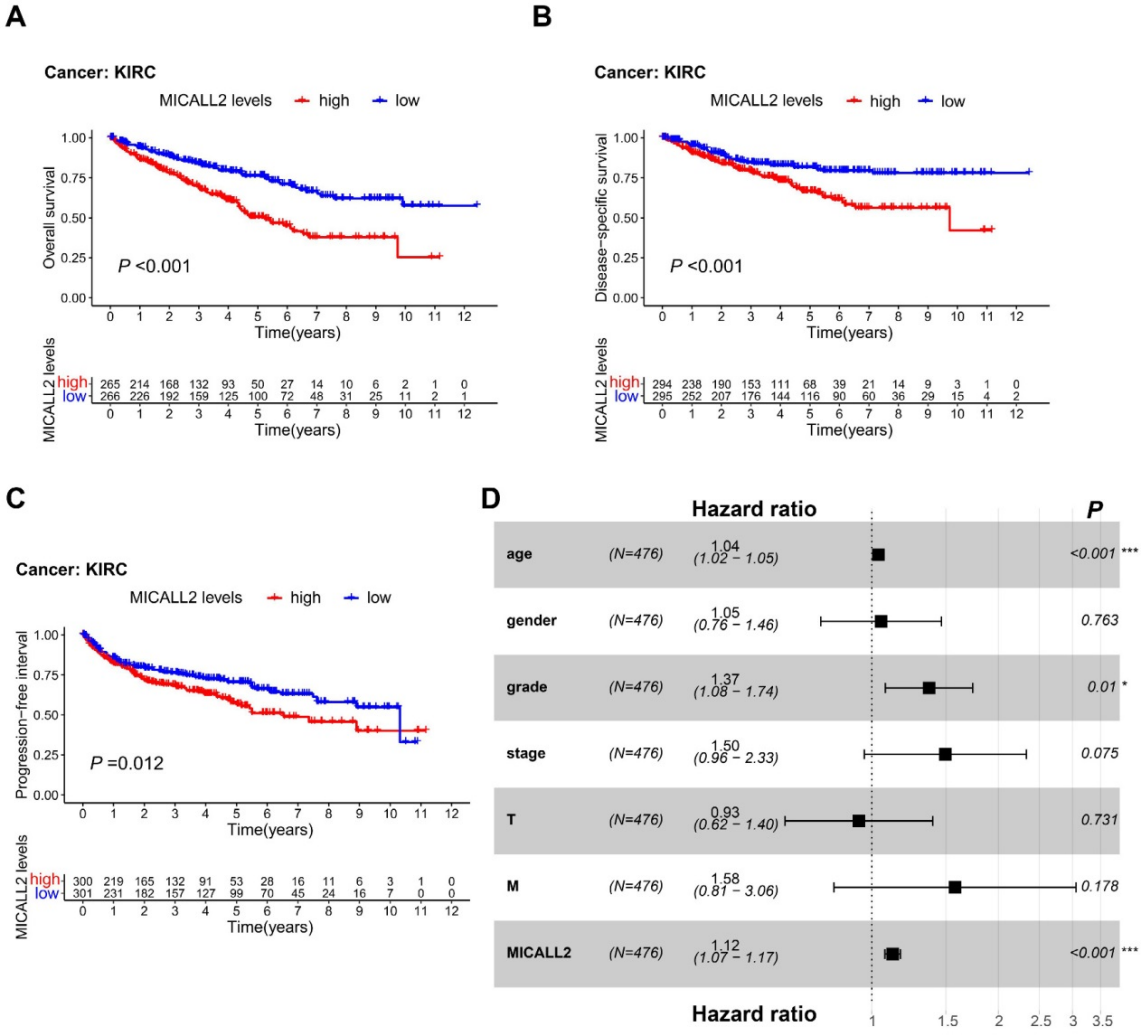
** Prognostic value of MICALL2 in KIRC patients. (A-C)** K-M analysis of the association between MICALL2 levels and OS, DSS, and PFI in KIRC. **(D)** The forest plot showing multivariate Cox regression analysis of predictive factors including age, gender, pathological grade, clinical stage, T classification, M classification, and MICALL2 expression. (*P <0.05, **P <0.01, ***P <0.001). **Abbreviations:** K-M, Kaplan-Meier; PFI, Progression-Free Interval; DSS, Disease-Specific Survival; OS, overall survival.

To reveal the association between MICALL2 levels and prognosis in KIRC patients, we performed K-M survival analyses, finding that KIRC patients with lower MICALL2 levels had a longer OS, DSS, and PFI (**Figure 4A-4C**) compared with those with higher MICALL2 levels. Cox regression was further applied for the impact assessment of clinicopathological factors and MICALL2 expression on survival based on univariate and multivariate analyses. In univariate analysis, the significant predictors of survival included age (hazard ratio, HR, 1.03; 95% confidence interval, 95% CI, 1.02-1.04; P <0.001), pathological grade (HR, 2.24; 95% CI, 1.82-2.76; P <0.001), clinical stage (HR, 1.88; 95% CI, 1.64-2.15; P <0.001), T classification (HR, 1.90; 95% CI, 1.60-2.25; P <0.001), M classification (HR, 4.40; 95% CI, 3.21-6.05; P <0.001), and MICALL2 expression (HR, 1.16; 95% CI, 1.11-1.20; P <0.001) (**Table S1**). Furthermore, these clinicopathological factors and MICALL2 expression were included in multivariate analysis, showing that age (HR, 1.04; 95% CI, 1.02-1.05; P <0.001), pathological grade (HR, 1.37; 95% CI, 1.08-1.74; P =0.01), and MICALL2 expression (HR, 1.12; 95% CI, 1.07-1.17; P <0.001) were important independent predictors for poor prognosis in KIRC patients (**Table S1, Figure 4D**).

### MICALL2 correlates with the majority of co-expressed immunoregulatory genes

**Figure 5:**
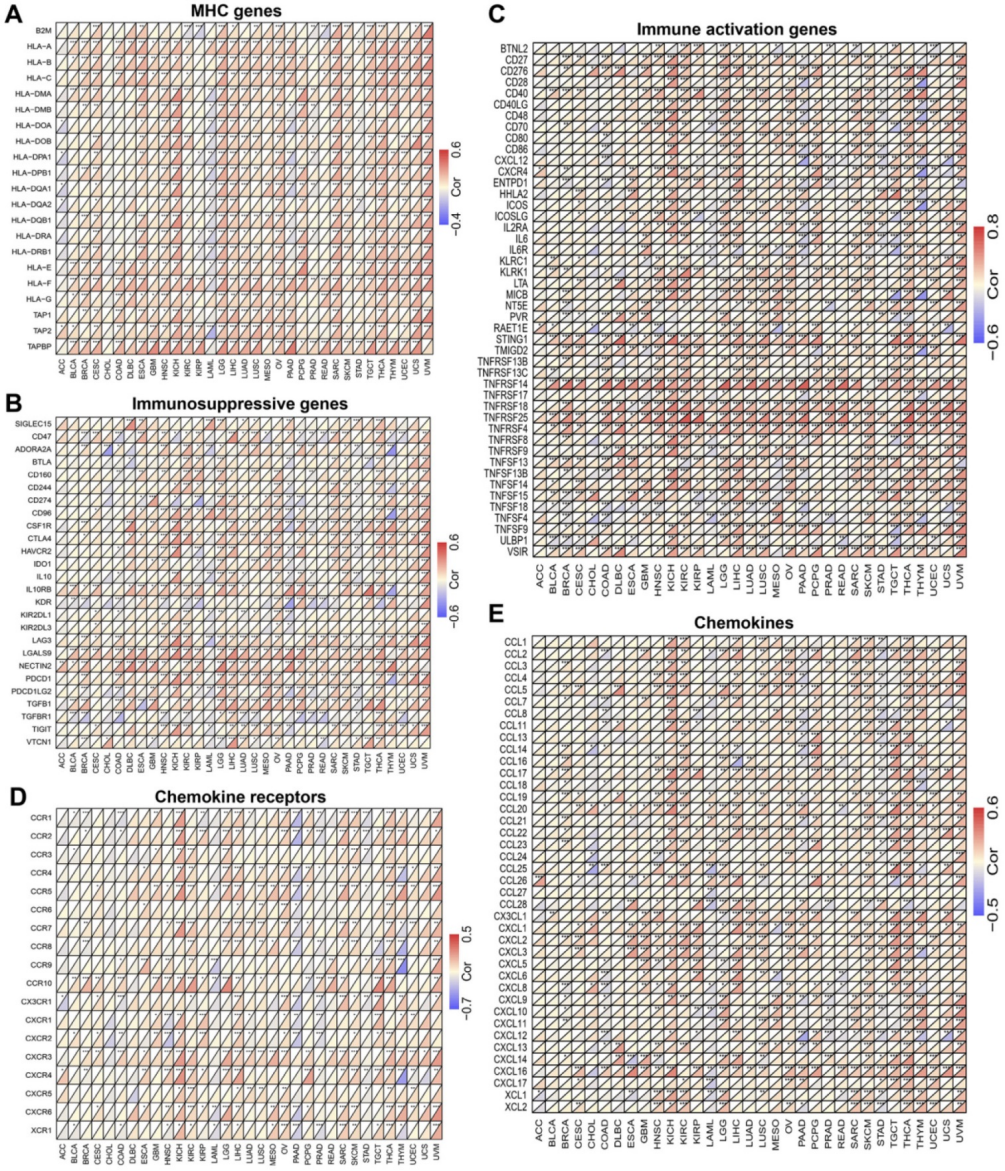
** The co-expression analysis of MICALL2 and immunoregulatory genes in pan-cancer.** The heatmaps presenting the correlations of MICALL2 expression with immunoregulatory genes related to **(A)** MHC, **(B)** immunosuppression, **(C)** immune activation, **(D)** chemokine receptors, and **(E)** chemokines. (*P <0.05, **P <0.01, ***P <0.001). **Abbreviations:** MHC, major histocompatibility complex.

**Figure 6:**
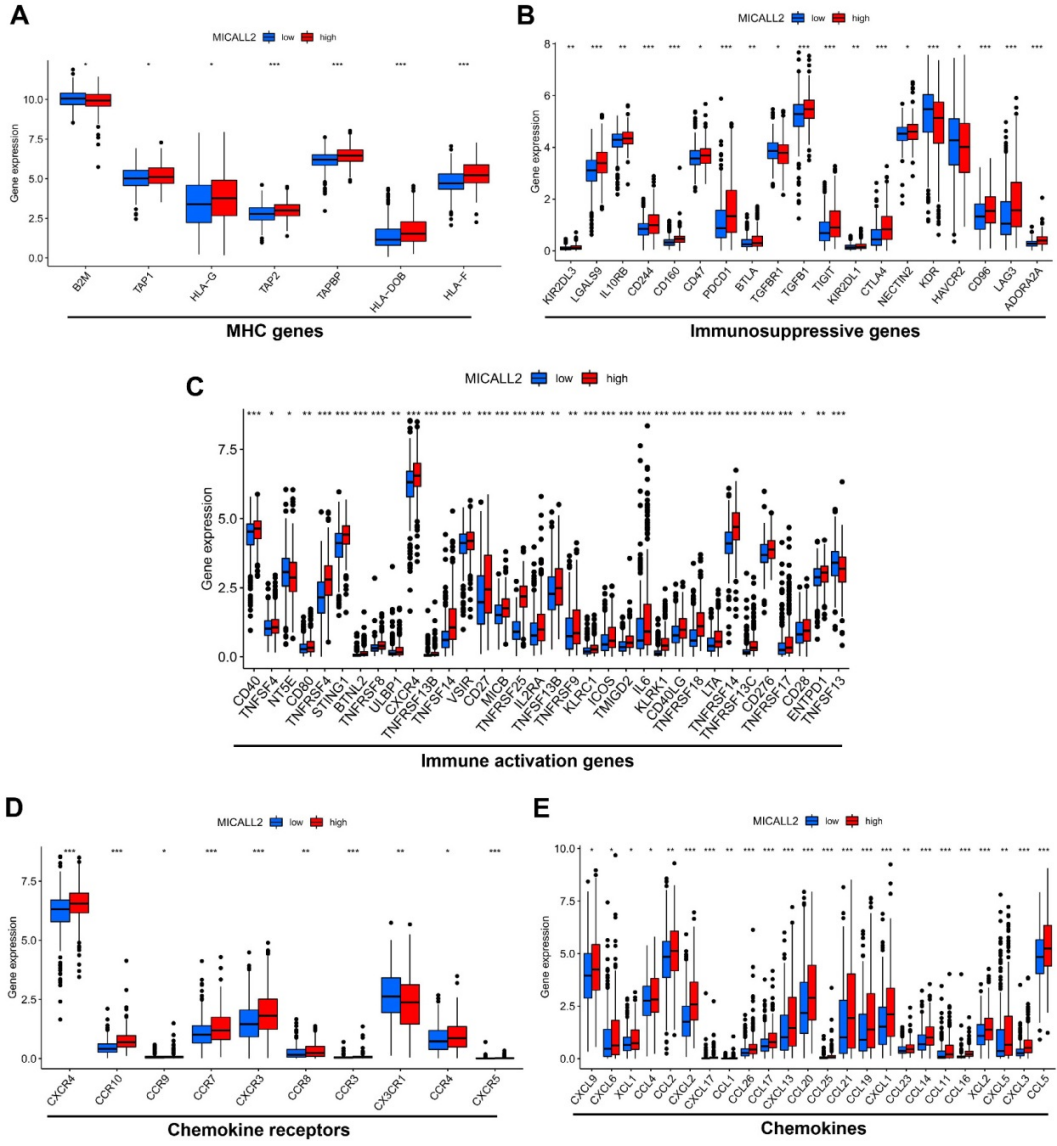
** The expression difference of immunoregulatory genes between high-MICALL2 and low-MICALL2 KIRC.** The box plots showing that those immunoregulatory genes with significantly different expressions between high-MICALL2 group and low-MICALL2 group, including** (A)** MHC genes, **(B)** immunosuppressive genes, **(C)** immune activation genes, **(D)** chemokine receptors, and **(E)** chemokines. (*P <0.05, **P <0.01, ***P <0.001).

To explore the role of MICALL2 in immunoregulation, we firstly performed a pan-cancer co-expression analysis on the immunoregulatory genes encoding MHC, immunosuppression, immune activation, chemokine receptors, and chemokines proteins (**Table S2**). Among the co-expressed immunoregulatory genes, the majority were positively correlated with MICALL2 (**Figure 5A-5E**). Furthermore, the heatmaps also revealed the positive correlation between MICALL2 and T cell exhaustion markers, such as programmed cell death protein 1 (PD-1/PDCD1), CD160, cytotoxic T-lymphocyte-associated protein 4 (CTLA-4), lymphocyte activation gene 3 (LAG3), and T-cell immunoglobulin and immunoreceptor tyrosine-based inhibitory motif (ITIM) domain (TIGIT). Therefore, we further analyzed the expression difference of immunoregulatory genes between high-MICALL2 and low-MICALL2 KIRC. Among the immunoregulatory genes with significantly different expressions, the majority, such as T cell exhaustion markers, had a higher expression in high-MICALL2 group compared with low-MICALL2 group (**Figure 6A-6E**).

### MICALL2 is a critical factor to influence immune infiltration in the TME

**Figure 7:**
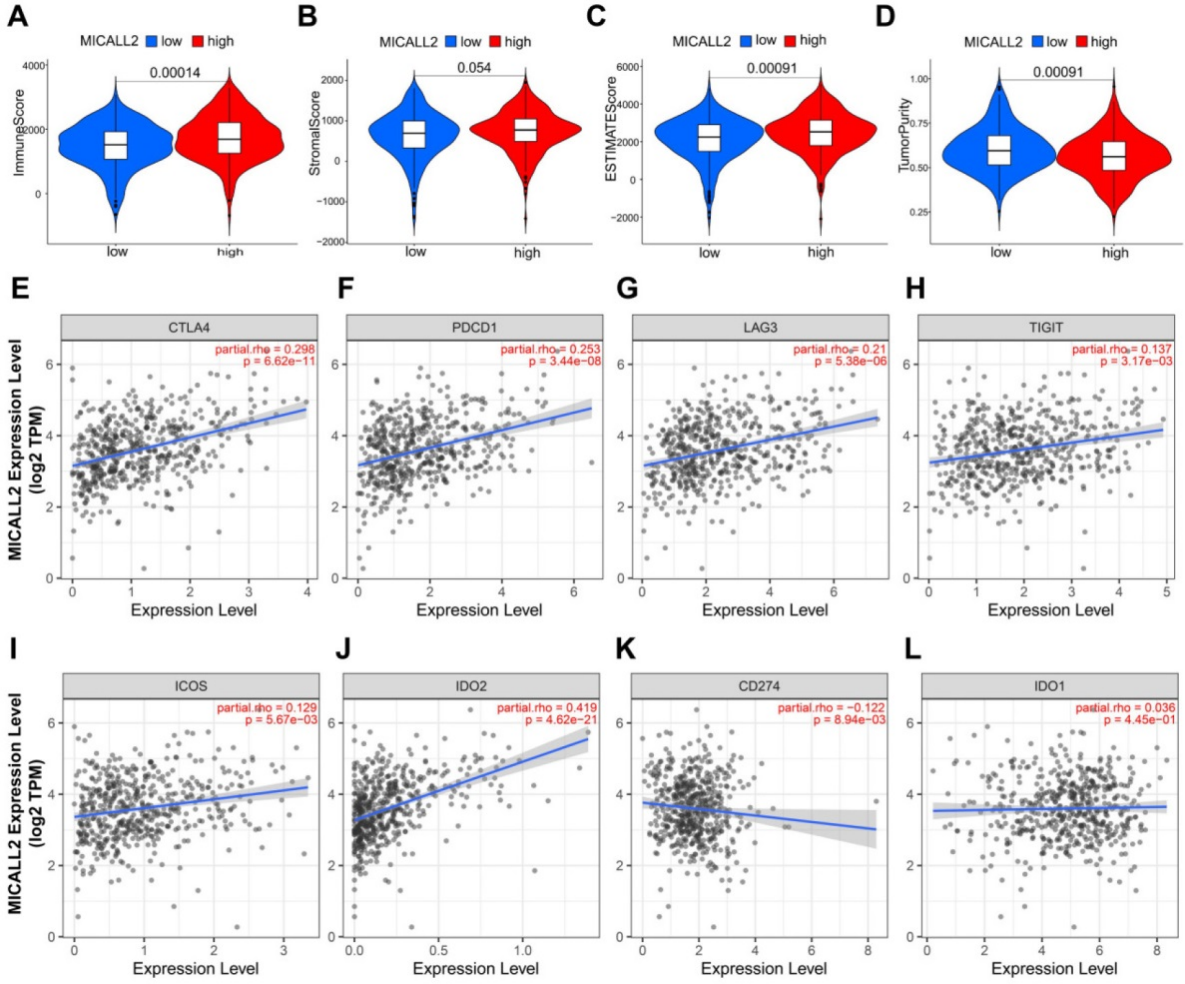
** Association of MICALL2 with the TME Composition and checkpoint gene expression.** Immune score, **(B)** Stromal score, **(C)** ESTIMATE score, and **(D)** Tumor purity were analyzed through ESTIMATE algorithm calculation to reveal the immune and stromal composition in TME. **(E-L)** The correlation analysis between MICALL2 expression and the expression of the common checkpoint genes. **Abbreviations:** TME, tumor microenvironment.

**Figure 8:**
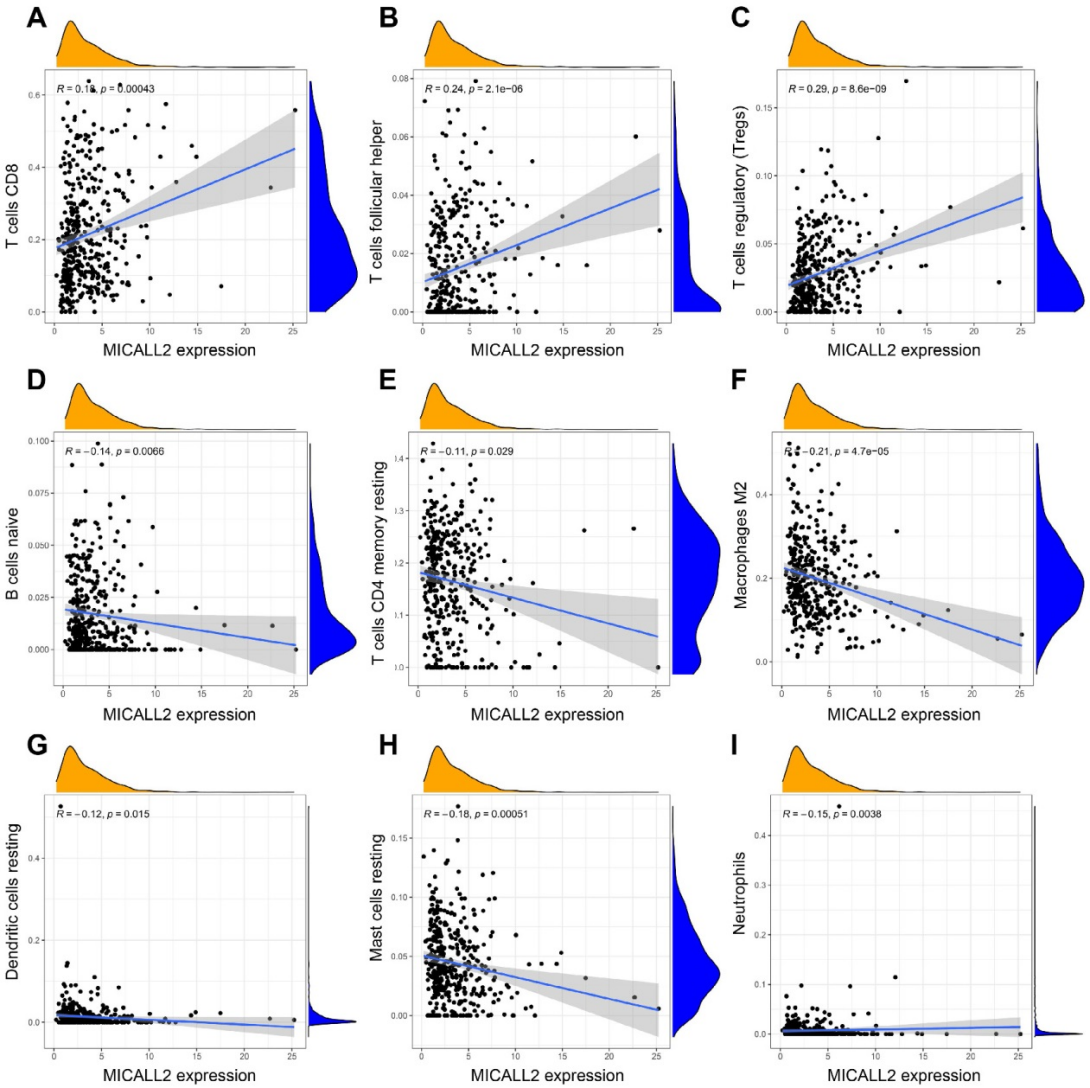
** Correlation of MICALL2 expression with TIICs in KIRC. (A-I)** Scatter plots showing that 9 kinds of TIICs were significantly correlated with MICALL2 expression. **Abbreviations:** TIICs, tumor-infiltrating immune cells.

The results in **Figure 7A-7D** revealed the immune and stromal compositions in the TME of KIRC. MICALL2 expression was positively correlated with Immune score (P <0.001), and ESTIMATE score (P <0.001). But it was negatively correlated with Tumor purity (P <0.001), and there was no significant difference in Stromal score (P =0.054). MICALL2 expression had a positive correlation with the expression of common checkpoint genes such as CTLA4, PDCD1, LAG3, TIGHT, inducible T cell costimulator (ICOS), and indoleamine 2,3-dioxygenase 2 (IDO2) (**Figure 7E-7J**). But there was a negative correlation between MICALL2 expression and programmed cell death ligand 1 (PD-L1/CD274) expression (**Figure 7K**), and no significant difference between MICALL2 expression and IDO1 expression (**Figure 7L**). Next, we applied the CIBERSORT tool to analyze the correlation of MICALL2 expression with TIICs (22 immune cell types). Among them, 3 kinds of TIICs were positively correlated with MICALL2 expression, including T cells regulatory (Tregs), T cells follicular helper, and T cells CD8, while 6 kinds of TIICs were negatively correlated with MICALL2 expression, including T cells CD4 memory resting, B cells naive, Macrophages M2, Dendritic cells resting, Mast cells resting, and Neutrophils (**Figure 8A-8I**).

**Figure 9:**
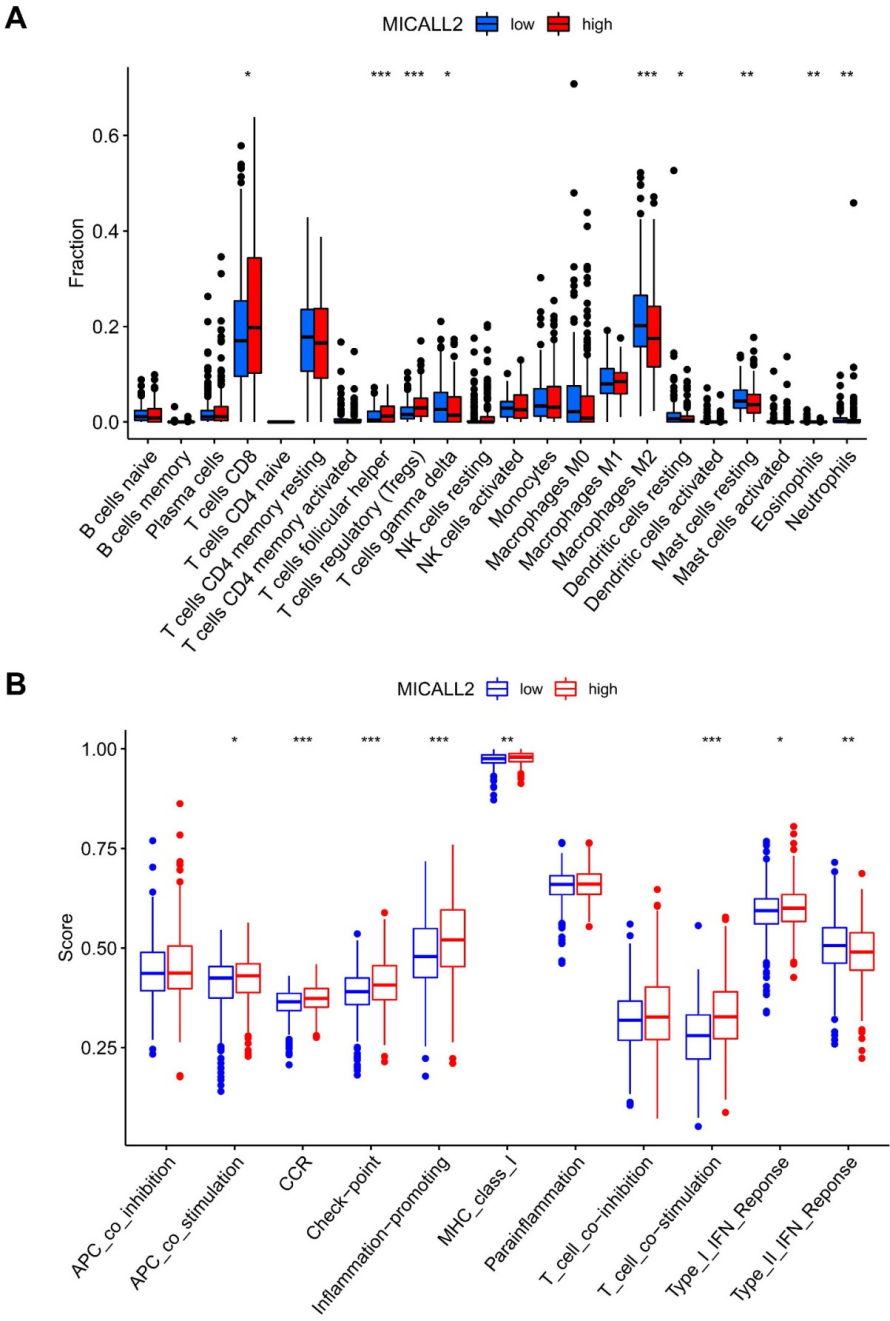
** The immune infiltration difference between high-MICALL2 and low-MICALL2 groups. (A)** The comparison of immune cell fraction (22 immune cell types). **(B)** The comparison of 11 immune signatures by ssGSEA algorithm. **Abbreviations:** ssGSEA, single sample Gene Set Enrichment Analysis.

We also assessed the ratio differentiation of 22 immune cell types. As shown in **Figure 9A**, the immune cell fractions of Tregs, T cells follicular helper, and T cells CD8 were higher in the high-MICALL2 group compared with the low-MICALL2 group. In contrast, the immune cell fractions of T cells gamma delta, Macrophages M2, Dendritic cells resting, Mast cells resting, Eosinophils, and Neutrophils were lower in the high-MICALL2 group than the low-MICALL2 group.

We further analyzed 11 immune signatures including pathways, checkpoints, and functions (**Table S3**). The immune signatures of MHC class I, APC co stimulation, CCR, checkpoint, inflammation-promoting, T cell co-stimulation, and Type I IFN response, had higher ssGSEA scores in the high-MICALL2 group than the low-MICALL2 group. In contrast, the ssGSEA score of Type II IFN response in the high-MICALL2 group was lower than that in the low-MICALL2 group (**Figure 9B**).

### MICALL2 regulates the signaling pathways related to tumor immunity, tumor progression, and cancer metabolism

**Figure 10:**
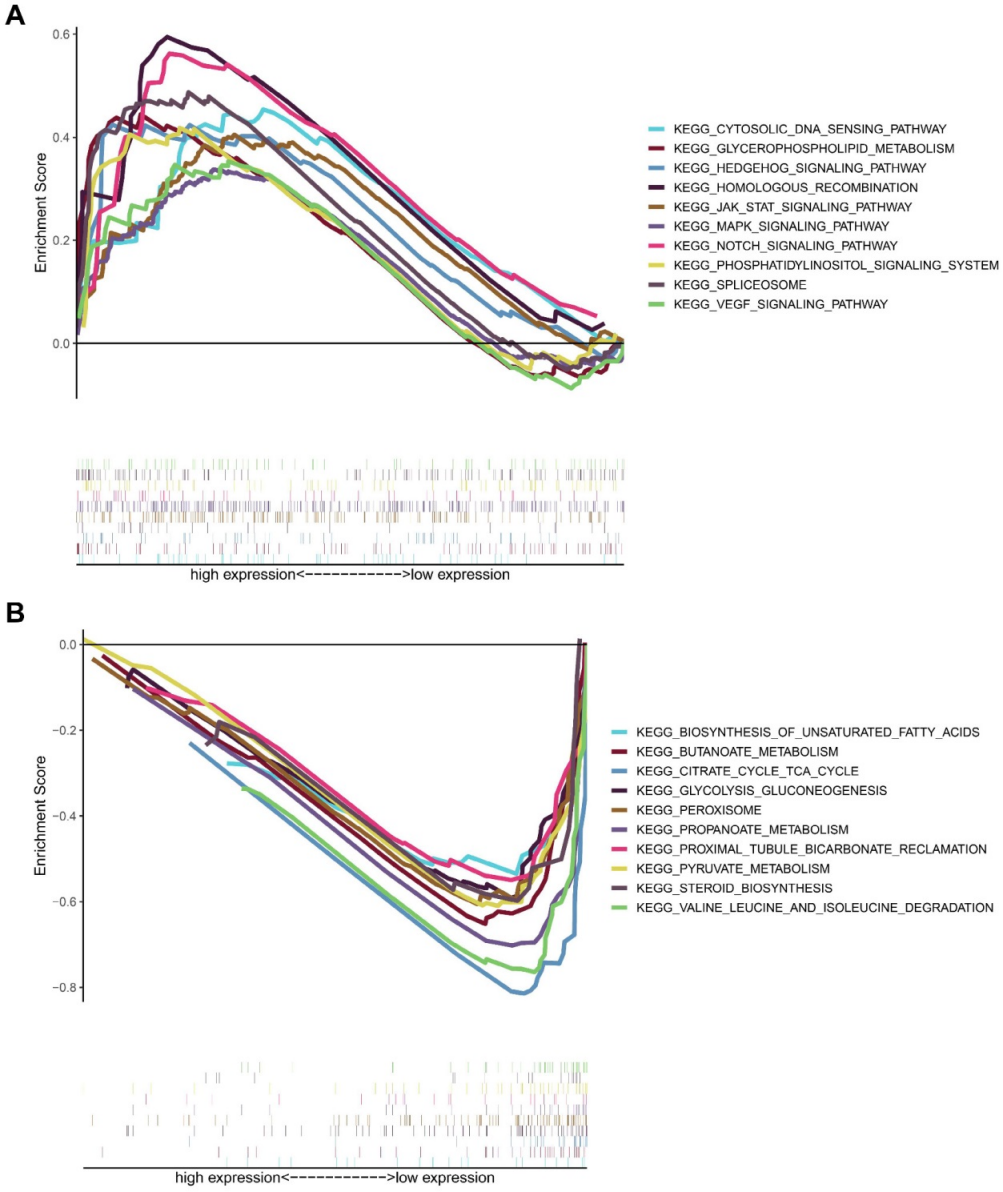
** KEGG enrichment analysis of MICALL2 in KIRC from TCGA dataset**. The enriched gene sets in MICALL2 high-expression group (nominal P <0.05). **(B)** The enriched gene sets in MICALL2 low-expression group (nominal P <0.05). Curves in different colors indicate different pathways or functions. **Abbreviations:** KEGG, Kyoto Encyclopedia of Genes and Genomes.

To analyze the biological significance of MICALL2, KIRC samples from TCGA database were divided into two groups. Then GSEA was introduced to assess those enriched KEGG pathways between these two groups. The genes in MICALL2 high-expression group were mainly enriched in Cytosolic DNA-sensing pathway, Glycerophospholipid metabolism, Hedgehog signaling pathway, Homologous recombination, MAPK signaling pathway, JAK-STAT signaling pathway, Notch signaling pathway, Phosphatidylinositol signaling system, Spliceosome, VEGF signaling pathway (**Figure 10A**). The genes in low-expression group were significantly enriched in metabolic pathways related to Biosynthesis of unsaturated fatty acids, Butanoate metabolism, Citrate cycle TCA cycle, Glycolysis/gluconeogenesis, peroxisome, Propanoate metabolism, Proximal tubule bicarbonate reclamation, Pyruvate metabolism, Steroid biosynthesis, Valine, leucine and isoleucine degradation (**Figure 10B**). These findings revealed that MICALL2 could involve in the regulation of signaling pathways associated with tumor immunity, tumor progression, and cancer metabolism.

### Biological functions and signaling pathway of the DEGs associated with MICALL2 expression

In the GSE53757 dataset, the patients were divided into high-MICALL2 group and low-MICALL2 group based on MICALL2 median expression level. As shown in the volcano map (**Figure S1A**), a total of 1299 DEGs were identified with 582 downregulated genes and 717 upregulated ones, of which the top 30 ones were visualized on the heatmap based on |logFC| values (**Figure S1B**). The GO enriched DEGs significantly focused on T cell activation, regulation of immune effector process, regulation of cell-cell adhesion, neutrophil activation, and positive regulation of cytokine production (**Figure S1C**). In KEGG pathway analysis, the DEGs mainly focused on chemokine signaling pathway, cytokine-cytokine receptor interaction, JAK-STAT signaling pathway, NF-kappa B signaling pathway, and cell adhesion molecules (**Figure S1D**).

## Discussion

The interplay of inflammation and immunity affects all aspects from tumorigenesis to progression, and even tumor response to therapy 22. The therapeutic prospect of renal cell carcinoma (RCC), a highly immunogenic and vascularized cancer type 23, has been recently revolutionized via immunotherapy stimulating the immune system and anti-angiogenesis therapy inhibiting RCC angiogenesis 24. However, RCC microenvironment, extensively characterizing angiogenesis, inflammatory, and immune signatures, exhibits the different responses to immune checkpoint blockade and anti-angiogenic therapeutics 25. It remains a challenge to explore predictive and prognostic biomarkers for existing regimens management and targeting drug development. The most common subtype KIRC, also known as clear cell RCC (ccRCC), accounts for 75-80% of RCC cases 26. For the first time, this present study integratedly assessed the effects of MICALL2 in patient prognosis, tumor progression, inflammatory, and immune signatures of KIRC.

In this study, we analyzed the expressions of MICALL2 across 33 cancer types, finding that compared with the corresponding normal tissues, MICALL2 was highly expressed in 16 types of cancers including KIRC while MICALL2 levels were downregulated in PCPG tissues. We also verified the high expression of MICALL2 in KIRC through GEPIA platform and GEO datasets. In accordance with the pan-cancer analysis, prior studies showed that MICALL2 is highly expressed in ovarian cancer, gastric cancer, and lung cancer demonstrated by western blot or immunohistochemical staining 17-19. In addition, MICALL2 expressions increased with higher pathological grade, clinical stage, T classification, and M classification. Consistently, Zhu et al. found that the highly expressed MICALL2 closely correlates with advanced clinicopathological parameters in ovarian cancer 17. These findings above indicated that MICALL2 could be an oncogene, which plays a key role in KIRC progression. Interestingly, the DEGs associated with MICALL2 expression were significantly enriched in functional regulation of cell-cell adhesion, and cell adhesion molecules, which are consistent with a previous study reporting MICALL2 regulation in the epithelial cell adhesion, repulsion, and even scattering 15, 16. Furthermore, MICALL2 may involve in the regulation of signaling pathways associated with cancer progression, such as JAK-STAT signaling pathway, MAPK signaling pathway, and VEGF signaling pathway. To date, no research has reported the association between MICALL2 and survival outcomes. According to our results, K-M method revealed that the KIRC patients with higher MICALL2 levels had a shorter OS, DSS, and PFI, while multivariate analysis suggested MICALL2 as an independent predictor for poor prognosis of KIRC patients.

In recent years, significant changes occur in KIRC immunotherapy, ranging from traditional immunoenhancement causing frequent immune-related adverse events by interferon alpha (IFN-α) and interleukin 2 (IL-2) to the more efficacious and less toxic immune normalization with programmed cell death 1 (PD-1) or cytotoxic T lymphocyte-associated antigen 4 (CTLA-4) antibodies in advanced KIRC 27. In this study, T cells CD8 were positively correlated with MICALL2 expression. Moreover, high-MICALL2 group possessed higher ssGSEA scores in the immune signatures of MHC class I, APC co stimulation, checkpoint, CCR, T cell co-stimulation, and Type I IFN response. However, researchers have revealed that T cells CD8 expressing T cell exhaustion markers cannot perform their usual function due to the persistent exposure to antigenic stimulation 28. One example of T cell exhaustion in KIRC is that TNFRSF9+ CD8+ T cells, exhibiting both effector and exhaustion phenotypes, are demonstrated as an adverse prognostic indicator 29. Our results showed the increased immunosuppressive subset of CD4+ T cells called Tregs 30, and the upregulated T cell exhaustion markers such as TNFRSF9, CD160, CTLA4, LAG3, and TIGIT, finally leading to immune escape and T cell dysfunction in advanced KIRC. The accumulation of T follicular helper cells (Tfh), a CD4+ T cell subset essential for B cell response shaping, can produce a negative or positive prognostic effect on multiple cancer types. A recent study has reported that the interaction of CD8+ exhausted T cells and Tfh plays a critical role in the anti-tumor immune response induced by anti-PD-L1/PD-1 immunotherapy 31. Our results showed a positive correlation between MICALL2 expression and Tfh, indicating that Tfh could also involve in the T cell exhaustion and affect the immunotherapeutic effect on KIRC. The DEGs associated with MICALL2 expression were significantly enriched in T cell activation, regulation of immune effector process, cytokine-cytokine receptor interaction, and chemokine signaling pathway.

Growing evidence suggests that chronic inflammation increases cancer risk, but their specific association remains unclear 22, 32. The inflammatory TME is a complex network composed of inflammatory cells, cytokines, chemokines, chemokine receptors, and signaling pathways 4. Our findings indicated high-MICALL2 group had higher ssGSEA scores in the immune signature of Inflammation-promoting. The molecular mechanisms to promote inflammation-mediated tumorigenesis by enrichment analysis were closely related to JAK-STAT signaling pathway, NF-κB signaling pathway, cytosolic DNA-sensing pathway, cytokine-cytokine receptor interaction, and chemokine signaling pathway, which have been summarized in prior studies 33, 34. Notably, increasing evidence has revealed the crucial interaction between JAK-STAT and NF-κB signaling pathway in developing inflammation-induced tumor 33. Simultaneous activation of STAT3 and NF-κB in stromal and tumor cells leads to the secretion of tumor-promoting factors, such as IL-6, and VEGF, triggering a positive feedback loop among inflammation, immunity, and tumorigenesis 35, 36. These findings above indicate that it could be a therapeutic strategy to normalize or remodel the inflammatory TME of KIRC by targeting the inflammatory factors or enriched pathways.

Unlike traditional Warburg effect, the subversive findings reveal that cellular programming drives immune cells and cancer cells to preferentially obtain glucose and glutamine, respectively 37. Therapeutic strategies targeting cell-selective nutrients partitioning can be developed to regulate the metabolic activities in specific cell populations of TME 38. Here we found the impaired metabolism pathways in both high-MICALL2 and low-MICALL2 groups, so more studies are expected to figure out the correlation between these metabolism pathways and specific cell populations.

To date, this is the first report to demonstrate that MICALL2 could be a prognostic biomarker associating with cancer progression, inflammatory, and immune signatures of KIRC. Due to the highly heterogeneous, adaptive, and dynamic TME, more basic and preclinical studies are expected to further verify the specific molecular mechanism of MICILL2 in regulating the inflammatory and immune functions, and the corresponding signaling pathways we enriched above. In addition, novel drugs targeting MICALL2 signaling should also be developed for the treatment of KIRC. It should be noted that, for KIRC, single agent therapy potentially causes drug resistance and limited therapeutic efficacy 39. Further clinical studies are required to test the combined efficacy of anti-MICALL2 therapy with immune checkpoint blockade or anti-angiogenic therapeutics.

## Conclusion

Here we reported for the first time that MICALL2 is highly expressed in multiple cancer types and can serve as an independent predictor for poor prognosis in KIRC patients. MICALL2 expression is correlated with inflammation-promoting response, immune cell infiltration, and T cell exhaustion within the microenvironment of KIRC. Furthermore, MICALL2 can regulate the signaling pathways related to tumor immunity, tumor progression, and cancer metabolism. Our findings could provide a molecular basis of MICALL2 as a prognostic biomarker for the precise and personalized treatment of KIRC.

## Supplementary Material

Supplementary figure and tables.

## Figures and Tables

**Table 1:** Characteristics of patients with KIRC

Characteristics	Variables	KIRC Cases (N=537)	Percentages (%)
Age	≤65	352	65.55
	>65	185	34.45
Gender	Male	346	64.43
	Female	191	35.57
Pathological grade	G1/G2	244	45.44
	G3/G4	285	53.07
	Unknown	8	1.49
Clinical stage	I/II	326	60.71
	III/IV	208	38.73
	Unknown	3	0.56
T classification	T1/T2	344	64.06
	T3/T4	193	35.94
M classification	M0	426	79.33
	M1	79	14.71
	Unknown	32	5.96
N classification	N0	240	44.69
	N1	17	3.17
	Unknown	280	52.14

**Abbreviations:** KIRC, kidney renal clear cell carcinoma.
