# Supplementary material for: Identification of MICALL2 as a Novel Prognostic Biomarker Correlating with Inflammation and T Cell Exhaustion of Kidney Renal Clear Cell Carcinoma
Source: J Cancer. 2022 Jan 16;13(4):1214–28. doi: 10.7150/jca.66922 (PMC8899381; doi:10.7150/jca.66922)
Supplement: Supplementary file 1 — Supplementary figure and tables. [file jcav13p1214s1.pdf]

Figure S1

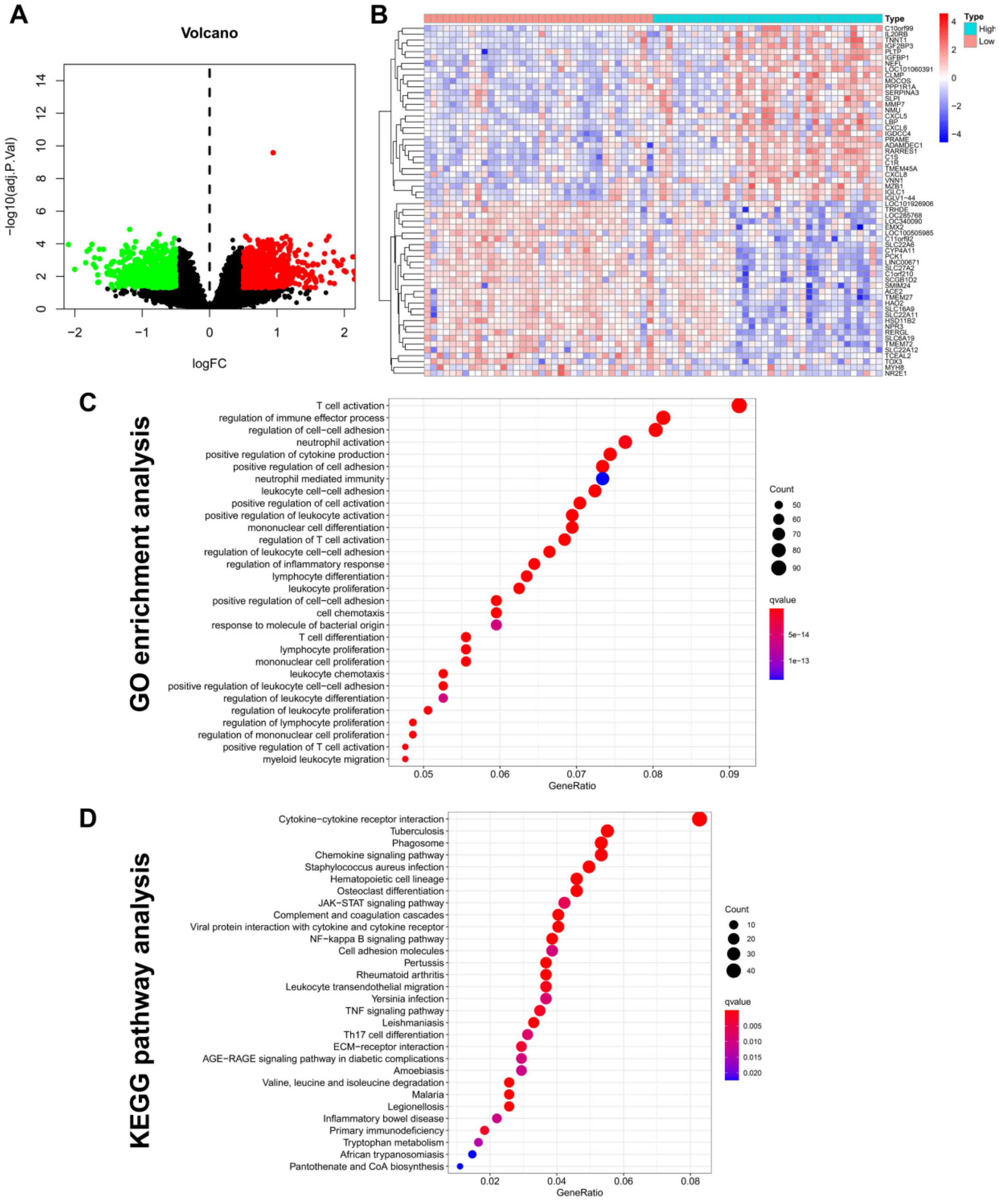

Figure S1 Enrichment analysis of DEGs between high-MICALL2 and low-MICALL2 expression samples from GEO dataset.

(A) Volcano map **(B)** Heatmap of DEGs between high-MICALL2 group and low-MICALL2 group in GSE53757. **(C)** The top 30 GO terms and **(D)** the top 30 KEGG pathways by enrichment analysis of the downregulated and upregulated genes. **Abbreviations:** GEO, Gene Expression Omnibus; GO, Gene Ontology; DEGs, differentially expressed genes.

Table S1 Univariate and multivariate Cox regression analysis for predictive factors

	Univariate analysis			Multivariate analysis		
**Parameters**	**HR**	**95%CI**	***P***	**HR**	**95%CI**	***P***
**age**	1.03	1.02-1.04	**0.000**	1.04	1.02-1.05	**0.000**
**gender**	0.94	0.69-1.30	0.726	1.05	0.76-1.46	0.763
**grade**	2.24	1.82-2.76	**0.000**	1.37	1.08-1.74	**0.010**
**stage**	1.88	1.64-2.15	**0.000**	1.50	0.96-2.33	0.075
**T**	1.90	1.60-2.25	**0.000**	0.93	0.62-1.40	0.731
**M**	4.40	3.21-6.05	**0.000**	1.58	0.81-3.06	0.178
**MICALL2**	1.16	1.11-1.20	**0.000**	1.12	1.07-1.17	**0.000**

Abbreviations: HR, hazard ratio; CI, confidence interval.

Table S2 The immunoregulatory genes applied for co-expression analysis

Immunoregulation	Gene set
**MHC**	TAPBP, TAP2, TAP1, HLA-G, HLA-F, HLA-E, HLA-DRB1, HLA-DRA, HLA-DQB1, HLA-DQA2, HLA-DQA1, HLA-DPB1, HLA-DPA1, HLA-DOB, HLA-DOA, HLA-DMB, HLA-DMA, HLA-C, HLA-B, HLA-A, B2M
**Immunosuppression**	VTCN1, TIGIT, TGFBR1, TGFB1, PDCD1LG2, PDCD1, NECTIN2, LGALS9, LAG3, KIR2DL3, KIR2DL1, KDR, IL10RB, IL10, IDO1, HAVCR2, CTLA4, CSF1R, CD96, CD274, CD244, CD160, BTLA, ADORA2A, CD47, SIGLEC15
**Immune activation**	VSIR, ULBP1, TNFSF9, TNFSF4, TNFSF18, TNFSF15, TNFSF14, TNFSF13B, TNFSF13, TNFRSF9, TNFRSF8, TNFRSF4, TNFRSF25, TNFRSF18, TNFRSF17, TNFRSF14, TNFRSF13C, TNFRSF13B, TMIGD2, STING1, RAET1E, PVR, NT5E, MICB, LTA, KLRK1, KLRC1, IL6R, IL6, IL2RA, ICOSLG, ICOS, HHLA2, ENTPD1, CXCR4, CXCL12, CD86, CD80, CD70, CD48, CD40LG, CD40, CD28, CD276, CD27, BTNL2
**Chemokine receptors**	XCR1, CXCR6, CXCR5, CXCR4, CXCR3, CXCR2, CXCR1, CX3CR1, CCR10, CCR9, CCR8, CCR7, CCR6, CCR5, CCR4, CCR3, CCR2, CCR1
**Chemokines**	XCL2, XCL1, CXCL17, CXCL16, CXCL14, CXCL13, CXCL12, CXCL11, CXCL10, CXCL9, CXCL8, CXCL6, CXCL5, CXCL3, CXCL2, CXCL1, CX3CL1, CCL28, CCL27, CCL26, CCL25, CCL24, CCL23, CCL22, CCL21, CCL20, CCL19, CCL18, CCL17, CCL16, CCL14, CCL13, CCL11, CCL8, CCL7, CCL5, CCL4, CCL3, CCL2, CCL1

Abbreviations: MHC, major histocompatibility complex.

Table S3 The comparison of 11 immune signatures by ssGSEA algorithm

Immune signatures	Gene set
**APC co inhibition**	C10orf54, CD274, LGALS9, PDCD1LG2, PVRL3
**APC co stimulation**	CD40, CD58, CD70, ICOSLG, SLAMF1, TNFSF14, TNFSF15, TNFSF18, TNFSF4, TNFSF8, TNFSF9
**CCR**	CCL16, TPO, TGFBR2, CXCL2, CCL14, TGFBR3, IL11RA, CCL11, IL4I1, IL33, CXCL12, CXCL10, BMPER, BMP8A, CXCL11, IL21R, IL17B, TNFRSF9, ILF2, CX3CR1, CCR8, TNFSF12, CSF3, TNFSF4, BMP3, CX3CL1, BMP5, CXCR2, TNFRSF10D, BMP2, CXCL14, CCL28, CXCL3, BMP6, CCL21, CXCL9, CCL23, IL6, TNFRSF18, IL17RD, IL17D, IL27, CCL7, IL1R1, CXCR4, CXCR2P1, TGFB1I1, IFNGR1, IL9R, IL1RAPL1, IL11, CSF1, IL20RA, IL25, TNFRSF4, IL18, ILF3, CCL20, TNFRSF12A, IL6ST, CXCL13, IL12B, TNFRSF8, IL6R, BMPR2, IFNE, IL1RAPL2, IL3RA, BMP4, CCL24, TNFSF13B, CCR4, IL2RA, IL32, TNFRSF10C, IL22RA1, BMPR1A, CXCR5, CXCR3, IFNA8, IL17REL, IFNB1, IFNAR1, TNFRSF1B, CCL17, IFNL1, IL16, IL1RL1, ILK, CCL25, ILDR2, CXCR1, IL36RN, IL34, TGFB1, IFNG, IL19, ILKAP, BMP2K, CCR10, ILDR1, EPO, CCR7, IL17C, IL23A, CCR5, IL7, EPOR, CCL13, IL2RG, IL31RA, TNFAIP6, IFNL2, BMP1, IL12RB1, TNFAIP8, IL4R, TNFRSF6B, TNFAIP8L1, TNFRSF10B, IFNL3, CCL5, CXCL6, CXCL1, CCR3, TNFSF11, CSF1R, IL21, IL1RAP, IL12RB2, CCL1, IL17RA, CCR1, IL1RN, TNFRSF11B, TNFRSF14, IL13, IL2RB, BMP8B, CCL2, IL24, IL18RAP, TGFBI, TNFSF10, TNFRSF11A, CXCL5, IL5RA, TNFSF9, IL1RL2, TNFRSF13C, IL36G, IL15RA, TNFRSF21, CXCL8, IL22RA2, TNFAIP8L2, IL18R1, IFNLR1, CXCR6, CCL3L3, TNFRSF1A, IL17RE, IFNGR2, IL17RC, TNFAIP8L3, ILVBL, TGFBRAP1, CCL4L1, CSF2RA, CCRN4L, CCL26, TNFAIP1, CCRL2, IFNA10, TNFRSF17, IFNA13, IL20, IL18BP, CCL3L1, TNFSF12-TNFSF13, IL5, IL23R, IL26, TNF, TGFA, CSF2, IL1F10, CXCL17, TNFSF13, IFNA4, IL37, IL12A, IL7R, IFNA1, IL1A, IL4, IL2, CCL22, CSF3R, IL10, IFNK, TGFB2, IL1R2, IL1B, IL17F, IL27RA, IL15, TNFSF8, IL36B, XCL1, CXCL16, TNFRSF19, IL3, CCL3, IFNA2, BMPR1B, IFNA21, TNFSF18, CCL8, IL17RB, TNFRSF25, IL22, IL10RB, IFNAR2, CCL18, IFNA16, CSF2RB, IL36A, TNFAIP3, IL13RA2, IL13RA1, CCR9, TNFRSF10A, IFNA7, IFNW1, XCL2, TNFSF14, CCR2, BMP15, BMP10, CCL15-CCL14, TGFBR1, IFNA5, BMP7, IFNA14, IL20RB, IL10RA, IFNA17, CCR6, TGFB3, CCL15, CCL4, CCL27, TNFRSF13B, TNFAIP2, IL31, IL17A, TNFSF15, CCL19, IFNA6, IL9
**Check-point**	IDO1, LAG3, CTLA4, TNFRSF9, ICOS, CD80, PDCD1LG2, TIGIT, CD70, TNFSF9, ICOSLG, KIR3DL1, CD86, PDCD1, LAIR1, TNFRSF8, TNFSF15, TNFRSF14, IDO2, CD276, CD40, TNFRSF4, TNFSF14, HHLA2, CD244, CD274, HAVCR2, CD27, BTLA, LGALS9, TMIGD2, CD28, CD48, TNFRSF25, CD40LG, ADORA2A, VTCN1, CD160, CD44, TNFSF18, TNFRSF18, BTNL2, C10orf54, CD200R1, TNFSF4, CD200, NRP1
**Inflammation-promoting**	CCL5, CD19, CD8B, CXCL10, CXCL13, CXCL9, GNLY, GZMB, IFNG, IL12A, IL12B, IRF1, PRF1, STAT1, TBX21
**MHC class I**	B2M, HLA-A, TAP1
**Parainflammation**	CXCL10, PLAT, CCND1, LGMN, PLAUR, AIM2, MMP7, ICAM1, MX2, CXCL9, ANXA1, TLR2, PLA2G2D, ITGA2, MX1, HMOX1, CD276, TIRAP, IL33, PTGES, TNFRSF12A, SCARB1, CD14, BLNK, IFIT3, RETNLB, IFIT2, ISG15, OAS2, REL, OAS3, CD44, PPARG, BST2, OAS1, NOX1, PLA2G2A, IFIT1, IFITM3, IL1RN
**T cell co-inhibition**	BTLA, C10orf54, CD160, CD244, CD274, CTLA4, HAVCR2, LAG3, LAIR1, TIGIT
**T cell co-stimulation**	CD2, CD226, CD27, CD28, CD40LG, ICOS, SLAMF1, TNFRSF18, TNFRSF25, TNFRSF4, TNFRSF8, TNFRSF9, TNFSF14
**Type I IFN Reponse**	DDX4, IFIT1, IFIT2, IFIT3, IRF7, ISG20, MX1, MX2, RSAD2, TNFSF10
**Type II IFN Reponse**	GPR146, SELP, AHR

Abbreviations: CCR, chemokine receptors.
